# One-year mortality risk in older individuals with femoral intertrochanteric fracture: a tertiary center in China

**DOI:** 10.1186/s12877-024-05159-y

**Published:** 2024-06-22

**Authors:** Youliang Hao, Ruideng Wang, Zhengyang Chen, Fang Zhou, Hongquan Ji, Yun Tian, Zhishan Zhang, Yan Guo, Yang Lv, Zhongwei Yang, Guojin Hou

**Affiliations:** 1 Department of Orthopaedics, Peking University Third Hospital, Beijing, 100191 China; 2 Engineering Research Center of Bone and Joint Precision Medicine, Beijing, 100191 China

**Keywords:** Mortality, Older individuals, Femoral intertrochanteric fracture, Risk factors

## Abstract

**Background:**

The accelerated growth of older individuals worldwide has increased the number of patients presenting with fragility hip fractures. Having a hip fracture can cause excess mortality, and patients with hip fracture have a higher risk of death than those without hip fracture. Most studies have treated hip fracture as a single, homogeneous condition, but hip fracture includes two major anatomic types: intertrochanteric fracture and femoral neck fracture. Few studies have specifically evaluated 1-year mortality risk in older individuals with femoral intertrochanteric fracture. The aim of this study was to evaluate 1-year mortality and factors associated with mortality in older individuals with femoral intertrochanteric fracture.

**Methods:**

A retrospective review was conducted of 563 patients ≥ 65 years old who underwent surgery for femoral intertrochanteric fractures at our institution between January 2010 and August 2018. Patient demographics, comorbidities, and treatment were collected by retrospective chart review. Age, sex, Body Mass Index (BMI), American Society of Anesthesiologists (ASA) classification, Charlson comorbidity index (CCI), Arbeitsgemeinschaft Für Osteosynthesefragen (AO) fracture classification, haemoglobin value at admission, time to surgery, operation time, and intraoperative blood loss were risk factors to be tested. Multivariable logistic regression was used to evaluate associations between variables and death.

**Results:**

Among the 563 patients, 49 died within 1 year after surgery, and the 1-year mortality rate was 8.7%. Multivariate analysis identified age > 80 years (OR = 4.038, *P* = 0.011), haemoglobin < 100 g/l (OR = 2.732, *P* = 0.002), ASA score ≥ 3 (OR = 2.551, *P* = 0.005), CCI ≥ 3 (OR = 18.412, *P* = 0.018) and time to surgery > 14 d (OR = 3.907, *P* = 0.030) as independent risk factors for 1-year mortality. Comorbidities such as myocardial infarction and chronic pulmonary disease were associated with 1-year mortality after adjusting for age > 80 years and time to surgery > 14 days.

**Conclusions:**

Patients over 80 years old with haemoglobin < 100 g/l, ASA score ≥ 3, CCI ≥ 3, and multiple comorbidities, especially myocardial infarction and chronic pulmonary disease before surgery, are at a higher risk of 1-year mortality. Doctors should pay more attention to these vulnerable patients, and a surgical delay greater than 14 days should be avoided.

## Background

Osteoporotic fractures pose an important economic and health burden in China and worldwide. Hip fracture is one of the most devastating consequences of osteoporosis [1] and is becoming one of the most important public health problems in the world [2]. Femoral intertrochanteric fracture and femoral neck fracture are its two main types. Although hip fracture is discussed as a unified entity, there are significant differences in the incidence rate and mortality after operation for femoral intertrochanteric fracture and femoral neck fracture. According to our previous systematic analysis, the pooled estimate of the 1-year mortality rate was 17.47% after femoral intertrochanteric fracture and 9.83% after femoral neck fracture between 2000 and 2018 [3]. Many studies [4–6] on hip fractures have described high mortality rates. Most studies document an increase in mortality lasting for 6 to 12 months [7, 8]. Identifying predictors of 1-year mortality could help discriminate between those at higher risk of adverse outcomes and facilitate targeted interventions [9]. Here we report the results of a consecutive population of femoral intertrochanteric fracture patients from our hospital. We reviewed the patient admission data and identified any predictors of patients at high risk of mortality not only during the immediate perioperative period but also over the year after discharge.

## Methods

### Patient data

The medical records of patients who underwent surgery for femoral intertrochanteric fractures at our institution between January 2010 and August 2018 were retrospectively reviewed. Inclusion criteria: (1) fresh closed femoral intertrochanteric fracture (≤ 3 weeks) and (2) age ≥ 65 years. Exclusion criteria: (1) pathological fracture, open fracture or periprosthetic fracture; and (2) poly-trauma.

### Operative protocol

Five experienced orthopaedic surgeons performed all of the surgeries. Spinal anaesthesia or general anaesthesia was used. Reduction and internal fixation were performed with the patients in the supine position on a fracture table using an image intensifier. After closed manipulation, intraoperative images were taken to evaluate the reduction quality of the fracture. Patients then underwent routine surgical procedures for internal fixation according to the manufacturer’s protocol.

### Time to mobilization after surgery

All patients commenced the routine rehabilitation treatment post-operation, which included immediate ankle pump training. On the first postoperative day, non-weight bearing joint mobility training was conducted, and patients were motivated to ambulate with assistance. Partial weight bearing was initiated once radiographic evidence of fracture healing appeared, progressing to full weight bearing upon clinical confirmation of fracture healing.

### Follow-up method

Patients were asked to return to hospital 1 month, 2 months, 3 months, 6 months, and 1 year after surgery. If the patients did not return on time, then a phone call or a video call was made to record their status. Patients who could not be reached after discharge were counted as lost to follow-up. Time to death (in months) was calculated from the date of surgery.

### Risk factors

The following general data were collected from medical records: age, sex, Body Mass Index (BMI), American Society of Anesthesiologists (ASA) classification, Charlson comorbidity index (CCI) [10] and age-adjusted CCI (aCCI) [11].

The following surgical data were also collected: the Arbeitsgemeinschaft Für Osteosynthesefragen (AO) classification [12] of the fracture, haemoglobin value (g/l) at admission, time to surgery (≤ 2 d, 2 d to 14 d, or > 14 d, time from injury to surgery), operation time (min), and intraoperative blood loss (ml).

### Statistical analysis

The chi-squared test was used to compare categorical variables between groups. For quantitative data, the one-sample Kolmogorov‒Smirnov test was used to test the normality of the distribution. Student’s t test or the Mann‒Whitney test was used to compare continuous variables, as appropriate. All variables were evaluated using unconditional univariate logistic regression analysis. Odds ratios (ORs) and 95% confidence intervals (CIs) were calculated. All variables with *P* < 0.05 in the univariate analysis were included in a multivariate model. Statistical significance was defined as *P* < 0.05. All statistical analyses were performed using SPSS 22.0 (SPSS, Chicago, IL, USA).

## Results

According to the inclusion criteria and exclusion criteria, 688 femoral intertrochanteric fractures were included in this study. There were 125 patients lost to follow-up. Therefore, a total of 563 patients were analysed. They were 175 (31.1%) males and 388 (68.9%) females. The patients were aged from 65 to 97 years, with an average of 80.1 ± 6.4 years. Among the 563 patients, 49 died within 1 year after surgery, for a 1-year mortality rate of 8.7%. Five patients died in hospital, for a nosocomial mortality rate of 0.9%.

The general and surgical data of living and dead patients were compared (Table 1). There were no significant differences in sex, BMI, AO classification, operation time, or intraoperative blood loss between groups (*P* > 0.05). The average age of patients in the deceased group was higher than that of patients in the alive group (*P* = 0.002). The 1-year mortality was higher in patients with ASA score ≥ 3 (51.0% versus 25.9% in survivors, *P* < 0.001). The results also showed differences in haemoglobin (*P* < 0.001), CCI (*P* = 0.07), aCCI (*P* = 0.016), and time to surgery (*P* = 0.029) between the two groups. To further analyse the differences between the two groups, a univariate analysis was conducted.

**Table 1:** Comparison of data between patients who survived and died at the 1-year follow-up

	Alive group (*n* = 514)	Mortality group (*n* = 49)	*P* value
**Age**	79.80 ± 6.39	82.78 ± 6.06	**0.002**
Sex (n,%)			0.804
Male	159 (30.9%)	16 (32.7%)	
Female	355 (69.1%)	33 (67.3%)	
BMI, Kg/m^2^ (n,%)			0.155
< 18.5	75 (14.6%)	12 (24.5%)	
18.5–23.9	249 (48.4%)	23 (46.9%)	
≥ 24.0	190 (37.0%)	14 (28.6%)	
**Haemoglobin, g/l (n,%)**			**<0.001**
< 100	121 (23.5%)	23 (46.9%)	
≥ 100	393 (76.5%)	26 (53.1%)	
**ASA (n,%)**			**<0.001**
1 to 2	381 (74.1%)	24 (49.0%)	
≥ 3	133 (25.9%)	25 (51.0%)	
**CCI (n,%)**			**0.007**
0	344 (66.9%)	28 (57.1%)	
1 to 2	162 (31.5%)	17 (34.7%)	
≥ 3	8 (1.6%)	4 (8.2%)	
**aCCI (n,%)**			**0.016**
≤ 3	152 (29.6%)	10 (20.4%)	
4 to 6	356 (69.3%)	36 (73.5%)	
≥ 7	6 (1.1%)	3 (6.1%)	
AO classification (n,%)			0.553
31A1	169 (32.9%)	15 (30.6%)	
31A2	292 (56.8%)	31 (63.3%)	
31A3	53 (10.3%)	3 (6.1%)	
**Time to surgery (n,%)**			**0.029**
≤ 2d	172 (33.5%)	10 (20.4%)	
2d to 14d	323 (62.8%)	34 (69.4%)	
> 14d	19 (3.7%)	5 (10.2%)	
Operation time (min)	62.0 (49.0 ~ 83.0)	60.0 (51.5 ~ 72.0)	0.725
Intraoperative blood loss (ml)	50 (50 ~ 100)	50 (50 ~ 100)	0.552

Abbreviations: *BMI* Body Mass Index; *ASA* American Society of Anesthesiologists; *CCI* Charlson Comorbidity Index; *aCCI* age-CCI; *AO* Arbeitsgemeinschaft Für Osteosynthesefragen

Values are presented as number of cases (%), mean ± SD or median (IQR)

Values in bold indicate *P* < 0.05, which is considered a significant difference and included in the multivariate regression analyses

Univariate analysis identified age > 80 years (*P* = 0.013), haemoglobin < 100 g/l (*P* = 0.001), ASA score ≥ 3 (*P* < 0.001), CCI ≥ 3 (*P* = 0.005), aCCI ≥ 7 (*P* = 0.009) and time to surgery > 14 d (*P* = 0.012) as risk factors for 1-year mortality (Table 2).

**Table 2:** Univariate and multivariate logistic regression analysis of risk factors for 1-year mortality

	Univariate analysis		Multivariate analysis	
	*P* value	OR (95% CI)	*P* value	OR (95% CI)
Age				
65 to 80 years		1.0 (reference)		1.0 (reference)
> 80 years	**0.013**	2.195 (1.179 ~ 4.087)	**0.011**	4.038 (1.377 ~ 11.844)
Haemoglobin				
< 100 g/l	**0.001**	2.873 (1.582 ~ 5.219)	**0.002**	2.732 (1.442 ~ 5.175)
≥ 100 g/l		1.0 (reference)		
ASA				
1 to 2		1.0 (reference)		1.0 (reference)
≥ 3	**<0.001**	2.984 (1.648 ~ 5.404)	**0.005**	2.551 (1.332 ~ 4.886)
CCI				
0		1.0 (reference)		1.0 (reference)
1 to 2	0.430	1.289 (0.686 ~ 2.423)	0.336	1.461 (0.675 ~ 3.161)
≥ 3	**0.005**	6.143 (1.742 ~ 21.667)	**0.018**	18.412 (1.636 ~ 207.218)
aCCI				
≤ 3		1.0 (reference)		1.0 (reference)
4 to 6	0.246	1.537 (0.744 ~ 3.176)	0.086	0.314 (0.084 ~ 1.178)
≥ 7	**0.009**	7.600 (1.651 ~ 34.975)	0.142	0.084 (0.003 ~ 2.289)
Time to surgery				
≤ 2d		1.0 (reference)		1.0 (reference)
2d to 14d	0.110	1.811 (0.873 ~ 3.753)	0.683	1.176 (0.541 ~ 2.554)
> 14d	**0.012**	4.526 (1.400 ~ 14.634)	**0.030**	3.907 (1.138 ~ 13.420)

Abbreviations: *ASA* American Society of Anesthesiologists; *CCI* Charlson Comorbidity Index; *aCCI* age-CCI; *OR* odds ratio; *CI* confidence interval

Values in bold indicate *P* < 0.05, which is considered a significant difference

After controlling for confounding variables, multivariate analysis identified age > 80 years (*P* = 0.011), haemoglobin < 100 g/l (*P* = 0.002), ASA score ≥ 3 (*P* = 0.005), CCI ≥ 3 (*P* = 0.018) and time to surgery > 14 d (*P* = 0.030) as independent risk factors for 1-year mortality (Table 2). In femoral intertrochanteric fracture patients, those over 80 years had a 4.038-fold higher risk of 1-year morality than the younger elderly. Patients with haemoglobin < 100 g/l had 2.732 times the risk of 1-year morality of those with haemoglobin ≥ 100 g/l. Patients with ASA score ≥ 3 had 2.551 times the risk of 1-year morality of those with a score < 3. Patients with CCI ≥ 3 had 18.412 times the risk of 1-year mortality of those with CCI = 0. Patients who underwent surgery more than 14 days after fracture had 3.907 times the risk of 1-year mortality of those who underwent surgery within 2 days.

Of the pathologies evaluated by the CCI, diabetes mellitus was the most common condition, being found in 17.1% (*n* = 96) of the cases, followed by cerebrovascular disease at 8.2% (*n* = 46), myocardial infarction at 5.5% (*n* = 31), any tumour at 3.0% (*n* = 17) and chronic pulmonary disease (chronic obstructive pulmonary disease (COPD) and asthma) at 2.0% (*n* = 11). Other diseases, such as dementia, connective tissue disease, renal disease, congestive heart failure, peripheral vascular disease, and metastatic solid tumours, were also present in the patients, but in fewer than 10 patients. Ulcer disease; mild, moderate or severe liver disease; leukaemia; lymphoma; and AIDS were not found in this study (Table 3).

**Table 3:** Association of comorbidities included in the CCI with 1-year mortality

Comorbidities	*n* (%)	Unadjusted OR (95% CI)	*P* value	Adjusted OR^*^ (95% CI)	*P* value
Myocardial infarction	31 (5.5)	2.729 (1.062 ~ 7.015)	**0.037**	2.705 (1.032 ~ 7.090)	**0.043**
Congestive heart failure	2 (0.4)	0	1	0	1
Peripheral vascular disease	2 (0.4)	0	1	0	1
Cerebrovascular disease	46 (8.2)	1.311 (0.493 ~ 3.488)	0.588	1.276 (0.467 ~ 3.434)	0.642
Dementia	9 (1.6)	0	1	0	1
Chronic pulmonary disease	11 (2.0)	4.058 (1.397 ~ 11.792)	**0.010**	3.815 (1.298 ~ 11.287)	**0.016**
Connective tissue disease	6 (1.1)	2.121 (0.243 ~ 18.525)	0.497	3.264 (0.356 ~ 29.948)	0.295
Diabetes mellitus	96 (17.1)	0.657 (0.272 ~ 1.591)	0.352	0.748 (0.305 ~ 1.835)	0.526
Hemiplegia	4 (0.7)	0	1	0	1
MS renal disease	6 (1.1)	5.426 (0.968 ~ 30.404)	0.054	4.625 (0.763 ~ 28.017)	0.096
Any tumour	17 (3.0)	1.416 (0.314 ~ 6.379)	0.651	1.207 (0.262 ~ 5.563)	0.809
Metastatic solid tumour	2 (0.4)	10.687 (0.658 ~ 173.578)	0.096	14.063 (0.713 ~ 277.473)	0.082

^*^ Adjusted for age > 80 years and time to surgery > 14 days

Abbreviations: *CCI* Charlson Comorbidity Index; *OR* odds ratio; *CI* confidence interval; *MS* moderate or severe

Values in bold indicate *P* < 0.05, which is considered a significant difference

Comorbidities such as myocardial infarction and chronic pulmonary disease were associated with mortality during the first post-operative year after adjusting for age > 80 years and time to surgery > 14 days (Table 3).

## Discussion

The accelerating growth of the global elderly population has increased the number of patients with fragile hip fractures [13, 14]. Hip fractures are among the fractures with the highest risk of death [15]. Hip fractures can lead to excess mortality, and patients with hip fractures have a higher risk of death than those without hip fractures [16, 17]. Results for 1-year mortality after hip fracture have been reported to vary. Abrahamsen et al. [18] performed a meta-analysis and found a 1-year mortality rate of 5.9 to 59%. In 2008 and 2009, the 1-year mortality rate after hip fractures among nursing home residents in Canada was approximately 45% [19]. Among Asian populations, the 1-year mortality rates were also different: 17.8% for Korean women [20], 18.65% for Hong Kong [21], and 13.5% for Taiwanese women [22]. In mainland China, a study showed the 1-year mortality in Beijing was approximately 23.44% [17]. Most studies have treated hip fracture as a single, uniform condition, but it includes two major anatomic types: intertrochanteric fractures and femoral neck fractures. The former is an extracapsular fracture, and the latter is an intracapsular fracture. A previous study showed that the 90-day mortality rate after intertrochanteric fracture of the femur was 12.1% and 9.6% after femoral neck fracture [23]. Another study showed that fracture type is an independent predictor of mortality in patients with hip fractures at 1 month and 1 year after injury [24]. Therefore, we should treat intertrochanteric fractures and femoral neck fractures differently and analyse their mortality rates separately.

In the present study of 563 patients who underwent surgery for femoral intertrochanteric fractures, 49 (8.7%) died within one year of surgery. Age, haemoglobin value at admission, ASA score, CCI and time to surgery were associated with 1-year mortality after surgery.

The present study showed that individuals over 80 years had a risk of 1-year morality 4.038 times that of younger individuals. Advanced age and male sex are known risk factors for mortality after hip fracture [25, 26]. Most previous studies have emphasized advanced age as the main cause of mortality and morbidity after hip fracture [27–29], but the relationship between sex and mortality remains controversial. Mariconda et al. [27] found that the 1-year mortality rate after a hip fracture was lower for patients aged > 80 years than for those aged < 80 years. Kim et al. [30] also found that age over 80 years was a predictor of 1-year mortality. Ferris et al. [8] found that male sex significantly predicted increased mortality, while other authors found that sex has no effect on mortality [29, 30]. In the present study, age was a significant predictor of 1-year mortality, but sex was not.

Nutritional status stands as a potential factor linked to 1-year mortality. Prior research has established a correlation between a low BMI and increased mortality rates [31]. Specifically, one study revealed that individuals with a BMI below 22 kg/m2 faced an almost seven-fold increase in 1-year mortality compared to those with a BMI exceeding 25 kg/m2 [32]. Another study echoed these findings, noting that obese patients (with a BMI of 30 kg/m2 or higher) exhibited the lowest risk of 1-year mortality, whereas those with a BMI less than 22 kg/m2 demonstrated the highest risk [33]. In our current study, although BMI served as a proxy for patients’ nutritional status, our analysis did not reveal a statistically significant difference in BMI between the survivors and the deceased (*P* > 0.05). Consequently, the impact of BMI on 1-year mortality warrants further examination.

Hip fracture patients, particularly those suffering from intertrochanteric fractures, frequently exhibit comorbid anemia. However, the degree of anemia and its association with 1-year mortality remain unclear. Espinosa et al. [34] found that haemoglobin values at the moment of hospital admission were associated with increased mortality, and the study showed that patients with haemoglobin < 10 g/dl had an increase of 1.51 times the mortality at 1 year, as compared to those with haemoglobin ≥ 10 g/dl. The present study showed same outcome, individuals with haemoglobin < 100 g/l had a risk of 1-year morality 2.732 times those haemoglobin ≥ 100 g/l. The result suggests that we should pay attention to the adverse effects of anemia, especially in individuals with haemoglobin less than 100 g/l.

The ASA score is based on a subjective assessment of patients’ health status before surgery. It was evaluated immediately prior to surgery. Liu et al. [14] found that ASA grading was significantly correlated with the incidence of postoperative complications and 1-year mortality in nonagenarians undergoing hip fracture surgery. Daugaard et al. [35] showed that increasing ASA score would significantly increase the mortality rate. Pugely et al. [36] also found that age greater than 80 years, male sex, declining functional status, ASA grade 3 or 4, and a history of cancer were independent risk factors for 30-day mortality. As in other studies [37–39], increasing ASA grade has independent deleterious effects on mortality and complications. This is consistent with the findings of our study, where an ASA score ≥ 3 was strongly associated with mortality. Patients with an ASA score ≥ 3 had a risk of 1-year mortality 2.551 times that of patients with an ASA score < 3.

Many studies have shown that comorbidities are associated with increased mortality or poor functional outcomes [16, 25, 29]. The CCI was developed by Charlson and colleagues in 1987 to classify comorbidities that may affect the risk of death [10]. The CCI considers 19 medical conditions, with the resulting score rising with their severity. The CCI is the most widely used comorbidity index for predicting survival rate (1 year and 10 years) in patients with multiple comorbidities. We hypothesized that multiple comorbidities (CCI ≥ 3) would be associated with a higher mortality, which was supported by our results. Patients with CCI ≥ 3 had a 18.412-fold risk of 1-year morality compared to those with CCI = 0.

In the present study, among various kinds of comorbidities, myocardial infarction and chronic pulmonary disease were found to be associated with 1-year mortality. These findings were mostly consistent with published papers. Espinosa et al. [29] found that diseases such as myocardial infarction, chronic pulmonary disease and hemiplegia caused by stroke were associated with increased 1-year mortality. Chang et al. [28] found that malignant tumor, cardiovascular disease and pulmonary disease were risk factors for mortality after hip fracture surgery. Friesendorff et al. [40] found that cardiovascular disease and pneumonia were the leading causes of death in both men and women of all age groups. Specifically, both myocardial infarction and cardiac failure are considered to be common determinants of death after hip fracture surgery [41]. Another study reported that patients with COPD had a 60–70% higher risk of death after hip fracture surgery than those without COPD [42]. In short, common comorbidities were associated with a higher risk of death after hip fracture surgery. The speculated reason is that patients with high mortality are often affected by postoperative complications such as infection, myocardial ischaemia, and thromboembolism. Given these findings, patients with these comorbidities require extra attention to reduce the risk of postoperative death.

Clinical guidelines recommend that elderly patients receive surgery within 48 h after hospital admission [43, 44]. However, in developing countries, there may be greater uncertainty about the effects of early surgery, as some studies have shown that postoperative mortality is similar in patients with short and long preoperative delays [29]. In contrast, in developed countries, patients may be admitted to hospital within 6 h after the trauma and receive surgical treatment within 24 h after the fracture, with similar mortality rates at one year after surgery [45]. In the present study, patients who underwent surgery after 14 days had a risk of 1-year mortality 3.907 times that of patients who underwent surgery within 2 days. However, the difference in 1-year mortality was not significant between patients who underwent surgery after 2 days but within 14 days and patients who underwent surgery within 2 days. In fact, immediate surgery can reduce immobilization time, the risk of bedsores, infections and thromboembolic complications. Some delays in surgery may be due to the severity of the patient’s health condition, which requires adequate treatment. A delay of more than 14 days should be avoided.

This study has several limitations. First, it was a retrospective observational study, and some patients were lost to follow-up. Second, it was a single-centre study, so there might have been selection bias. Third, some patients died in the community or other medical institutions, and we could not obtain the accurate causes of death from their family members, so the cause of death was not analyzed in this study.

## Conclusions

This study identified risk factors for 1-year mortality in older individuals with femoral intertrochanteric fracture. Patients over 80 years old with haemoglobin < 100 g/l, ASA score ≥ 3, CCI ≥ 3, and multiple comorbidities, especially myocardial infarction and chronic pulmonary disease before surgery, are at a higher risk of 1-year mortality. These results help identify vulnerable patients whom doctors should pay more attention to. As time to surgery is another factor associated with 1-year mortality, a surgical delay greater than 14 days should be avoided.

## Data Availability

The datasets analysed during the current study are available from the corresponding author on reasonable request.

## Abbreviations

BMI: Body Mass Index
ASA: American Society of Anesthesiologists
CCI: Charlson comorbidity index
AO: Arbeitsgemeinschaft Für Osteosynthesefragen
OR: odds ratio
CI: confidence interval
COPD: chronic obstructive pulmonary disease
